# Supplementary material for: Molecular insights into ago-allosteric modulation at cysteinyl leukotriene receptor 2
Source: Nat Commun. 2025 Dec 16;17:908. doi: 10.1038/s41467-025-67630-7 (PMC12830604; doi:10.1038/s41467-025-67630-7)
Supplement: Supplementary file 1 — Supplementary Information [file 41467_2025_67630_MOESM1_ESM.pdf]

Supplementary information

Molecular insights into ago-allosteric modulation at Cysteinyl leukotriene receptor 2

Mu Li[1#], Xiaoling Bao[2#], Wanbiao Chen[3#], Yusheng Guo[1#], Xiaomin Mao[1#], Miaofang Xiao[1#], Siqi Liu[1], Jiawei Li[3,4], Limin Zhao[4], Tiancai Chang[1], Fumei Zhong[1], Chongyuan Wang[3*], Heng Liu[1*]

[1]The Affiliated Traditional Chinese Medicine Hospital, GMU-GIBH Joint School of Life Sciences, The Guangdong-Hong Kong-Macao Joint Laboratory for Cell Fate Regulation and Diseases, Guangzhou Medical University, Guangzhou Municipal and Guangdong Provincial Key Laboratory of Protein Modification and Disease, State Key Laboratory of Respiratory Disease, 511436, Guangzhou, Guangdong, China.

[2]Scientific Research Center of Guangzhou Medical University, 511436, Guangzhou, Guangdong, China.

[3]Center for Human Tissues and Organs Degeneration, Faculty of Pharmaceutical Sciences, Shenzhen Institutes of Advanced Technology, Chinese Academy of Sciences, 581055, Shenzhen, Guangdong, China.

[4]Department of Geriatric Medicine, Shenzhen Longhua District Central Hospital, 518110, Shenzhen, Guangdong, China.

[#]These authors contributed equally.

*Correspondence: 2022991053@gzhmu.edu.cn (H.L.), cy.wang@siat.ac.cn (C.W.)

This file includes:

Supplementary Figures 1 to 6
Supplementary Tables 1 to 4

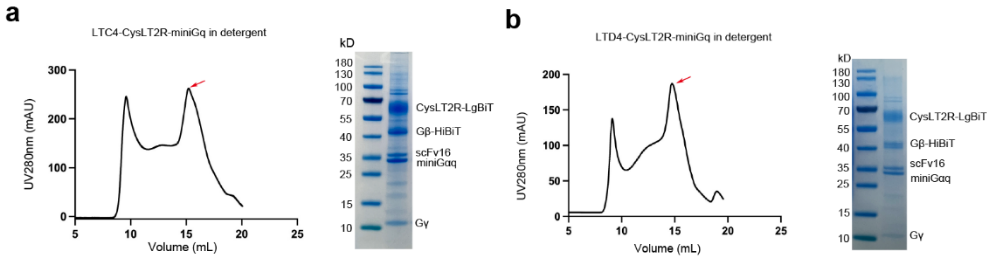

Supplementary Fig. 1: **Results of purification of LTC4- and LTD4- bound CysLT2R-miniG$_q$ complexes.** Representative elution profile on Superose 6 Increase 10/30 column and SDS–PAGE of the size-exclusion chromatography peak of LTC4-(**a**) and LTD4-(**b**) bound CysLT2R-miniG$_q$. Uncropped gels are provided in the Source Data file.

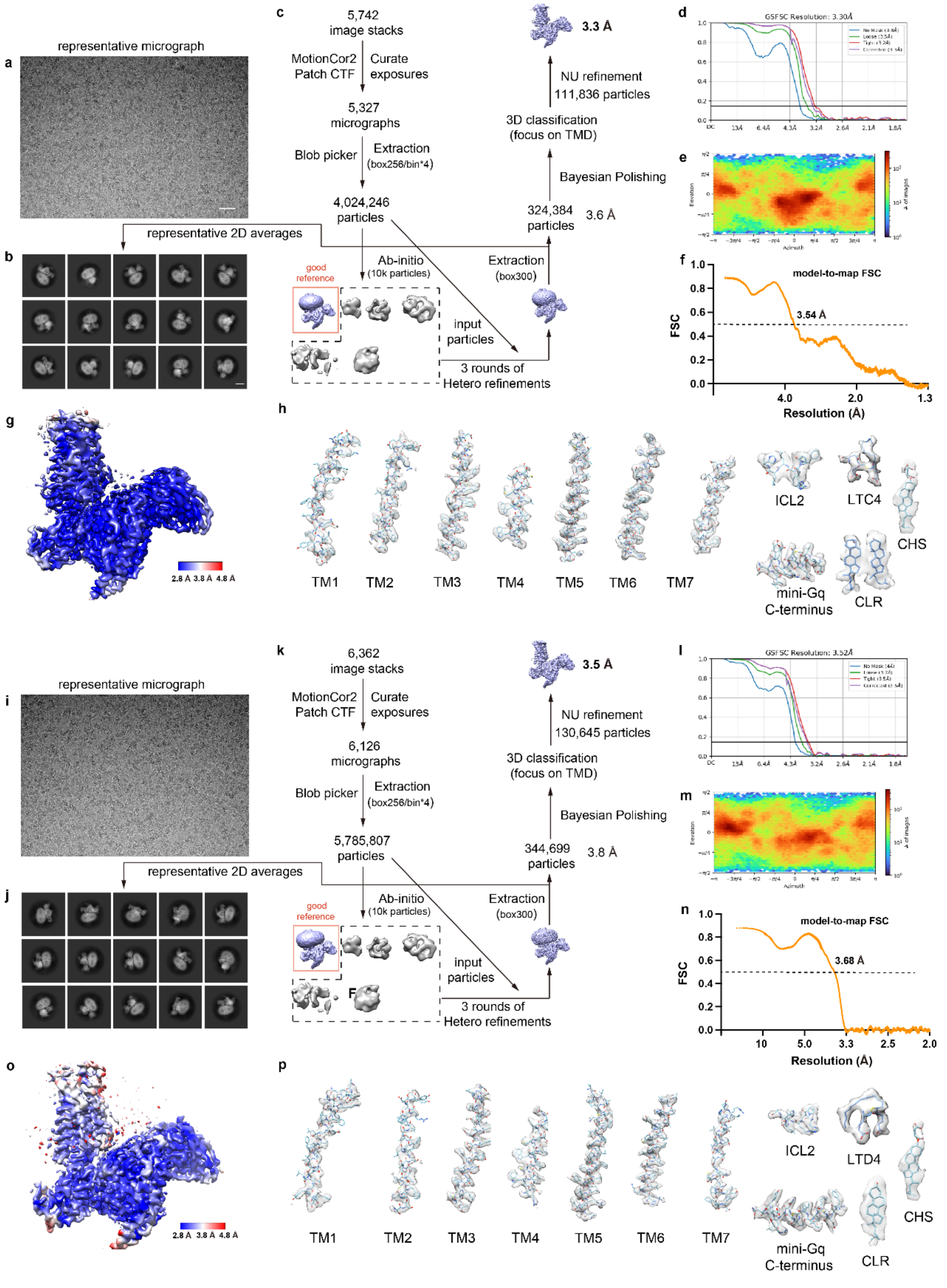

Supplementary Fig. 2: Cryo-EM data processing and reconstruction of CysLT2R-LTC4-miniG$_q$ (a-h) and CysLT2R-LTD4-miniG$_q$ (i-p). (**a**, **i**) Representative cryo-EM micrographs (scale bar: 50 nm). (**b**, **j**) Representative 2D class averages (scale bar: 5 nm). (**c**, **k**) Flow charts

outlining the cryo-EM processing workflows. (**d**, **l**) Half-map FSC curves. (**e**, **m**) the angular distributions Estimations of particles. (**f**, **n**) Model to map FSC curves. (**g**, **o**) Local resolution of the final reconstructions. (**h**, **p**) Models fit in maps. Details can be found in Methods.

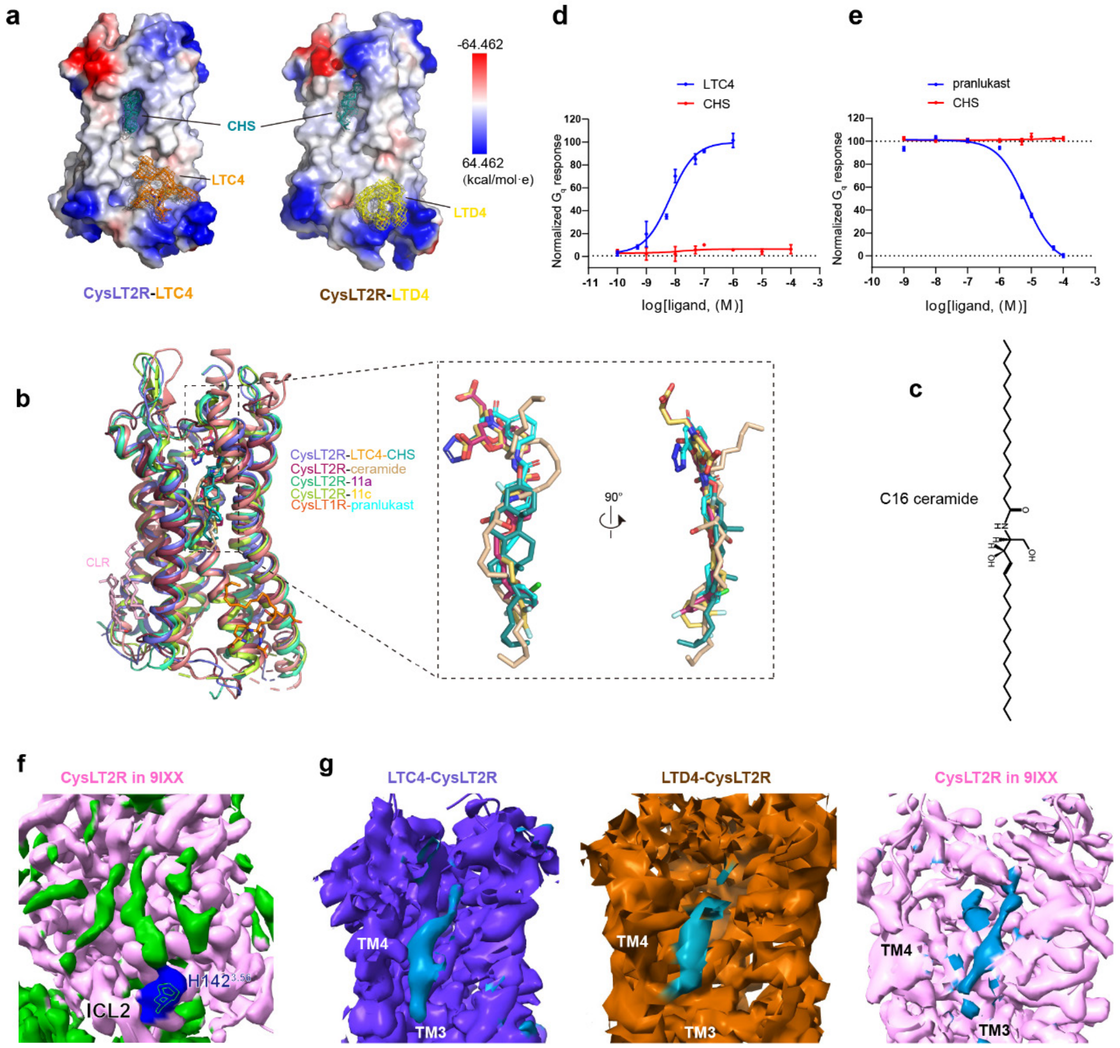

Supplementary Fig. 3: Multiple binding modes of different ligands in CysLT2R.

a Electrostatic surface potential of LTC4- and LTD4-bound CysLT2R.

b Superimposition of the structures of CysLT2R-LTC4, CysLT2R-C16:0 ceramide (PDB ID: 9JH5), CysLT2R-11a (PDB ID: 6RZ6), CysLT2R-11c (PDB ID: 6RZ8) and CysLT1R-pranlukast (PDB ID: 6RZ4). The ligands binding poses at TM4-TM5 cleft are shown in right panel.

c Chemical structure of C16:0 ceramide.

d, **e** Effects of CHS on CysLT2R activity in NanoBiT assays. (**d**) the activation of CysLT2R by LTC4 and CHS. (**e**) the inhibition of CysLT2R activity by antagonist pranlukast and CHS. All

data represent mean ± s.e.m. from three independent experiments (n=3). Source data are provided in the Source Data file.

f Electron densities (green) observed at allosteric pocket of CysLT2R (9IXX). The cryo-EM map is contoured at the level of 0.01.

g Electron densities (teal) observed at orthosteric pockets of CysLT2R-LTC4, CysLT2R-LTD4 and CysLT2R (9IXX). The cryo-EM map is contoured at the level of 0.14, 0.10 and 0.01, respectively.

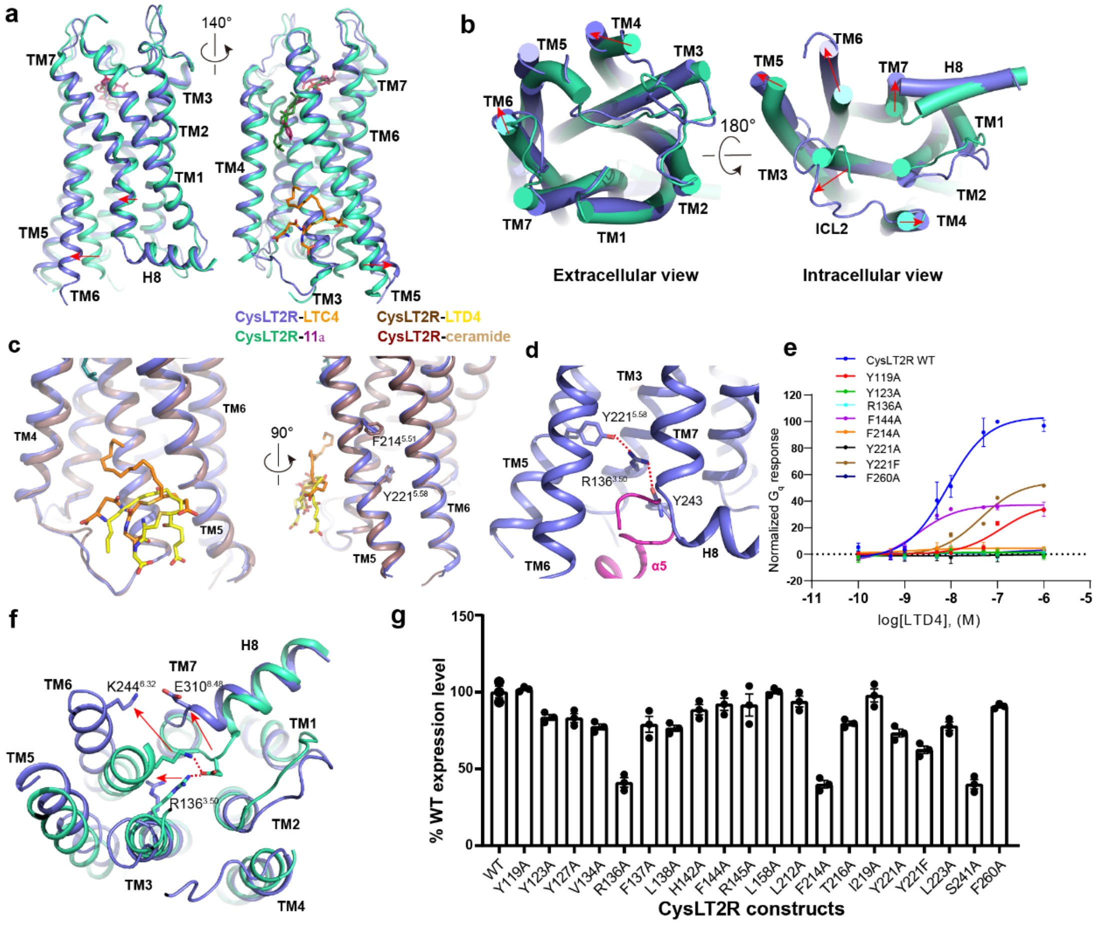

Supplementary Fig. 4: The conformational changes of CysLT2R during activation.

a, **b** Structure alignment of LTC4 activated CysLT2R and antagonist (11a) bound inactive CysLT2R (PDB ID: 6RZ6). (**a**): side view; (**b**): left: extracellular view; right: intracellular view. The conformational changes of active state compared with inactive state are marked with red arrows.

c the comparison of TM5 and TM6 conformation in LTC4- and LTD4-bound CysLT2R.

d Extensive polar interaction formed by R136$^{3.50}$, Y221$^{5.58}$ of CysLT2R and Y243 of Gα_q.

e Mutagenesis data stimulated by LTD4 using NanoBiT assays. All data represent mean ± s.e.m. from three independent experiments (n=3). Source data are provided in the Source Data file.

f The disruption of polar interaction network at cytoplasmic cavity upon activation. The

hydrogen bonds are shown as red dashed lines. The movement of residues from inactive state to active state is highlighted with red arrows.

g Measurement of the cell surface expression level of wild-type and mutants CysLT2R by FACS. Data are shown as the mean ± s.e.m. from three independent measurements (n=3). Source data are provided in the Source Data file.

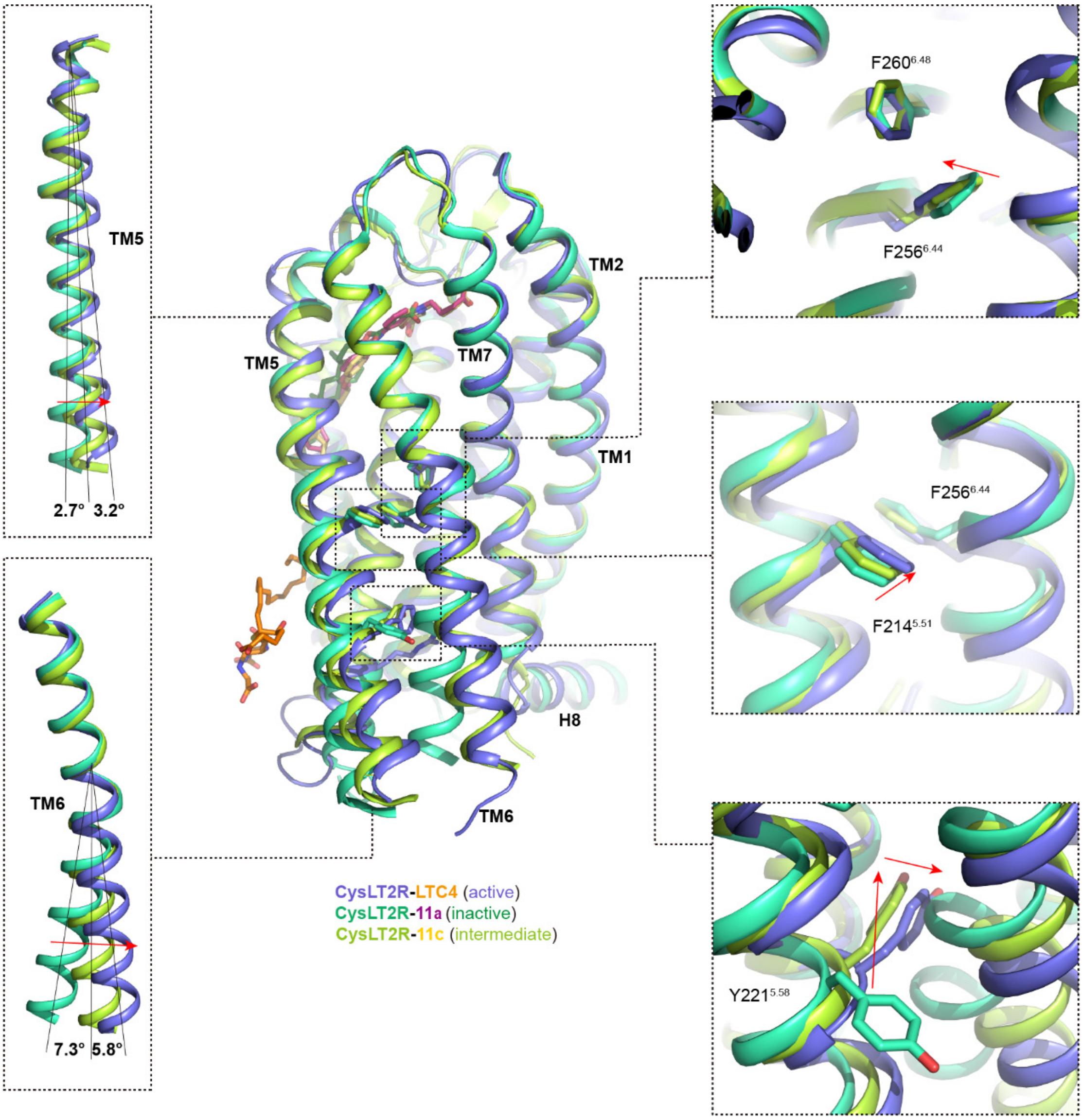

Supplementary Fig. 5: Conformational change trajectory during CysLT2R activation. The conformational change path from inactive to active state revealed by an intermediate 11c-bound CysLT2R structure (PDB ID: 6RZ8). The conformational change paths at different regions are indicated by red arrows.

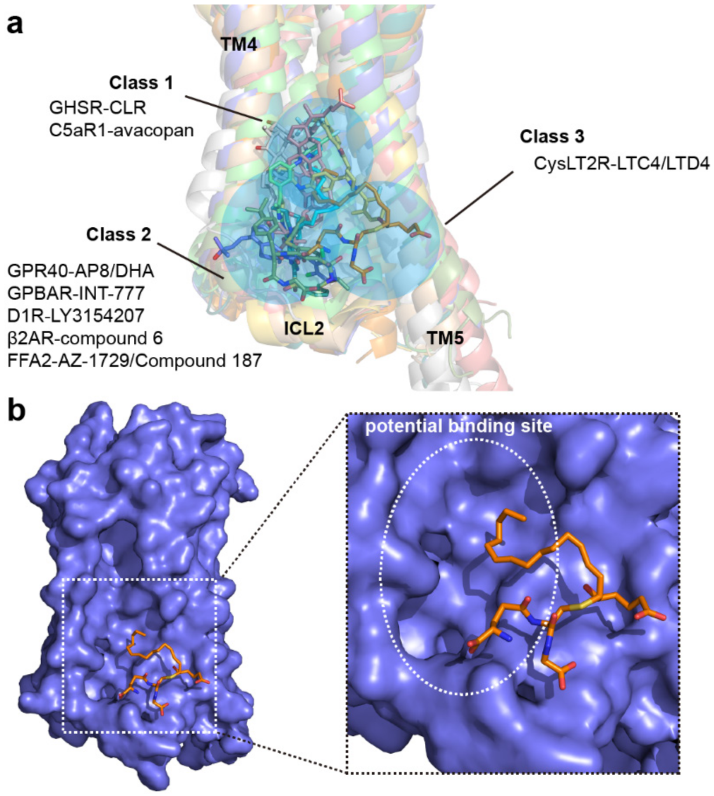

Supplementary Fig. 6: The multiple binding poses of ligands at "ICL2 site".

a Superimposition of GPCRs bound with molecules at "ICL2 site". Class 1: GHSR-CLR (PDB ID: 7NA7), C5aR1-avacopan (PDB ID: 6C1R); Class 2: GPR40-AP8/DHA (PDB ID: 5TZY, 9K1C), GPBAR-INT-777 (PDB ID: 7CFN), D1R-LY3154207 (PDB ID: 7X2F), β2AR-compound 6 (PDB ID: 6N48), FFA2-AZ-1729/compound 187 (PDB ID: 9CM7, 9CM3); Class 3： CysLT2R-LTC4/LTD4.

b A potential class 2 binding pocket at "ICL2 site" in CysLT2R.

Supplementary Table 1. Cryo-EM data collection, model refinement and validation statistics.

	CysLT2R-LTC4-G_q<br>PDB: 9UAM<br>EMD-63985	CysLT2R-LTD4-G_q<br>PDB: 9UAN<br>EMD-63986
**Data collection and processing**		
Microscope	FEI Titan Krios	FEI Titan Krios
Camera	Gatan K3	Gatan K3
Magnification	105,000×	105,000×
Voltage (kV)	300	300
Electron exposure (e^{-}/Å^2)	50	50
Defocus range (μm)	−1.0 ~ −2.3	−1.0 ~ −2.3
Pixel size (Å)	0.855	0.855
Software	RELION 4.1, CryoSPARC v4	RELION 4.1, CryoSPARC v4
Symmetry imposed	C1	C1
Initial particle images (no.)	4,024,246	5,785,807
Final particle images (no.)	324,384	130,645
Rsolution (FSC = 0.143, Å)	3.3	3.5
Local map resolution range (Å)	2.8-4.8	2.8-4.8
**Refinement**		
Software	Phenix 1.21real-space-refine	Phenix 1.21real-space-refine
Initial model (PDB code)	6RZ6	6RZ6
Model resolution (FSC=0.5/0.143, Å)	3.07/3.54	3.46/3.71

Map sharpening *B* factor (Å^2)	-90	-90
Model composition		
Non-hydrogen atoms	8,778	8,693
Protein residues (#)	1136	1136
Ligands	1(LTC4)/1(CHS)/2(CLR)	1(LTD4) 1(CHS)/1(CLR)
Water	0	0
B factors (Å^2)		
Protein	151.61	63.94
Ligand	159.10	73.04
R.m.s. deviations		
Bond lengths (Å)	0.003	0.004
Bond angles (°)	0.704	0.967
Validation		
MolProbity score	1.85	2.01
Clashscore	8.84	8.62
Poor rotamers (%)	3.55	0.77
Ramachandran plot		
Favored (%)	94.45	89.98
Allowed (%)	5.19	9.48
Disallowed (%)	0.36	0.54

Supplementary Table 2. Effects of CysLT2R mutations on LTC4 and LTD4 activity. NanoBiT-based assay was performed to evaluate G_q signals of CysLT2R induced by LTC4 and LTD4. Flow cytometry analyses were performed to evaluate the surface expression level of the CysLT2R mutants. Data are presented as mean ± s.e.m. of three independent experiments (n=3). ND indicates that the activation level is too low to detect. Source data are provided in the Source Data file.

Residue Number	CysLT2R mutations	LTC4		LTD4		Eexpression (%WT)
		pEC_{50} ± s.e.m.	E_{max} ± s.e.m. (%WT)	pEC_{50} ± s.e.m.	E_{max} ± s.e.m. (%WT)	
	WT	8.466 ± 0.05	100 ± 4.37	8.358 ± 0.10	100 ± 3.17	100 ± 3.84
3.33	Y119A	8.115 ± 0.09	48.34 ± 12.68	7.104 ± 0.68	34.24 ± 9.36	101.9 ± 0.92
3.37	Y123A	ND	2.869 ± 3.12	ND	ND	83.45 ± 1.91
3.41	Y127A	8.086 ± 0.11	20.17 ± 1.95	7.471 ± 0.9	13.87 ± 11.29	83.08 ± 2.94
3.48	V134A	8.674 ± 0.65	48.39 ± 17.16	7.686 ± 0.53	35.46 ± 5.01	77.33 ± 1.93
3.50	R136A	ND	ND	ND	ND	41.08 ± 3.03
3.51	F137A	8.253 ± 0.13	97.48 ± 9.45	7.875 ± 0.85	56.17 ± 5.41	78.96 ± 5.11
3.52	L138A	7.266 ± 0.25	42.76 ± 5.91	6.727 ± 0.92	43.93 ± 4.49	76.42 ± 2.01
ICL2	H142A	7.467 ± 0.41	29.55 ± 6.29	ND	8.06 ± 3.14	88.49 ± 3.33
ICL2	F144A	8.679 ± 0.33	48.64 ± 3.23	8.7 ± 0.48	36.1 ± 5.07	92.09 ± 4.00
ICL2	R145A	8.559 ± 0.09	90.07 ± 8	8.569 ± 0.25	74.39 ± 13.07	91.55 ± 7.17
4.45	L158A	7.31 ± 0.04	19.37 ± 1.69	6.786 ± 0.40	72.49 ± 23.73	100.2 ± 1.34
5.49	L212A	7.231 ± 0.01	53.07 ± 3.42	7.675 ± 0.18	14.62 ± 0.42	93.81 ± 3.46
5.51	F214A	6.704 ± 0.23	28.1 ± 2.84	ND	ND	39.89 ± 2.43
5.53	T216A	ND	6.576 ± 1.15	ND	4.99 ± 4.34	79.56 ± 1.54
5.56	I219A	8.361 ± 0.27	22.3 ± 5.12	ND	4.853 ± 6.25	97.8 ± 4.26
5.58	Y221A	ND	3.008 ± 0.97	ND	ND	73.26 ± 2.61
5.58	Y221F	7.393 ± 0.05	61.96 ± 3.58	7.369 ± 0.31	54.75	62.39 ± 2.44
5.6	L223A	8.201 ± 0.09	87.01 ± 5.34	8.219 ± 0.11	65.21 ± 3.59	77.79 ± 2.77
6.29	S241A	7.732 ± 0.13	58.62 ± 2.88	8.191 ± 0.19	52.35 ± 17.32	39.96 ± 2.98
6.48	F260A	8.11 ± 0.19	14.51 ± 4.30	ND	ND	90.8 ± 0.96

Supplementary Table 3. Enhanced effects of LTC4 on ceramide-stimulated CysLT2R signaling. Data represent concentration-response relationships of C16:0 ceramide-induced CysLT2R activation in the presence of increasing LTC4 concentrations. All data are shown as mean ± s.e.m. from at three independent (n=3) experiments performed in technical triplicate. Statistical significance (p value) was assessed by one-way ANOVA followed by Dunnett's multiple comparisons test, compared with the response of the C16:0 ceramide without LTC4. Source data are provided in the Source Data file.

LTC4, nM	CysLT2R activation on C16:0 ceramide			
	EC_{50} ± s.e.m. (μM)	pEC_{50} ± s.e.m.	ΔpEC_{50} ± s.e.m.	E_{max} ± s.e.m. % of efficacy at 0.1mM ceramide
0	1.27 ± 0.05	5.90 ± 0.02	0	100
0.01	0.88 ± 0.15	6.07 ± 0.07	0.17 ± 0.08 (p=0.8506)	111.3 ± 8.3
0.1	0.46 ± 0.0004	6.33 ± 0.0003	0.44 ± 0.02 (p=0.1201)	152.6 ± 14.9
1	0.21 ± 0.02	6.68 ± 0.05	0.78 ± 0.05 (p=0.0031)	194.4 ± 27.1
10	0.19 ± 0.01	6.73 ± 0.03	0.83 ± 0.04 (p=0.0019)	256.5 ± 33.1
100	0.15 ± 0.08	7.02 ± 0.32	1.12 ± 0.31 (p=0.0001)	391.7 ± 17.1
1000	0.005 ± 0.001	8.32 ± 0.101	2.42 ± 0.09 (p<0.0001)	452.8 ± 28.5

Supplementary Table 4. The List of CysLT2R Primers Sequences for Site-Direct Mutagenesis Studies.

Mutation site	Forward primer (5'-3')	Reverse primer (5'-3')
Y119A	TTCCTTGgctGTCAACATGTACAGCAGTATTTATTTCC	TGTTGACagcCAAGGAATAAGACATAATCCTGCAGG
Y123A	CAACATGgctAGCAGTATTTATTTCCTGACCGTGC	TACTGCTagcCATGTTGACATACAAGGAATAAGACATAA
Y127A	GCAGTATTgctTTCCTGACCGTGCTGAGTGTT	CAGGAAagcAATACTGCTGTACATGTTGACATACAAGG
V134A	CTGAGTgcaGTGCGTTTCCTGGCAATGGTTCA	AAACGCACtgcACTCAGCACGGTCAGGAAATAAA
R136A	TGTTGTGgctTTCCTGGCAATGGTTCACCCCT	CCAGGAAagcCACAACACTCAGCACGGTCAGG
F137A	AGTGTTGTGCGTgcaCTGGCAATGGTTCACCCCT	AGtgcACGCACAACACTCAGCACGGTCAGGAA
L138A	TGCGTTTCgcaGCAATGGTTCACCCCTTTCGG	CATTGCtgcGAAACGCACAACACTCAGCACGG
H142A	AATGGTTgcaCCCTTTCGGCTTCTGCATGTCA	GAAAGGGtgcAACCATTGCCAGGAAACGCACA
F144A	AATGGTTCACCCCgcaCGGCTTCTGCATGTCACCA	GtgcGGGGTGAACCATTGCCAGGAAACGCACA
R145A	ACCCCTTTgcaCTTCTGCATGTCACCAGCATCA	CAGAAGtgcAAAGGGGTGAACCATTGCCAGGA
L158A	TGGATCgcaTGTGGGATCATATGGATCCTTATCA	ATCCCACAtgcGATCCAGGCACTCCTGATGCT
L212A	CTGCCTGgcaCCATTTTTCACACTCAGCATCTG	AAAATGGtgcCAGGCAGCCCACCACCAAGGCA
F214A	TGCTGCCAgctTTCACACTCAGCATCTGTTATCTGC	TGTGAAagcTGGCAGCAGGCAGCCCACCACCA
T216A	CATTTTTCgcaCTCAGCATCTGTTATCTGCTGATCA	GCTGAGtgcGAAAAATGGCAGCAGGCAGCCCA
I219A	ACTCAGCgcaTGTTATCTGCTGATCATTCGGG	GATAACAtgcGCTGAGTGTGAAAAATGGCAGC
Y221A	CATCTGTgctCTGCTGATCATTCGGGTTCTGT	TCAGCAGagcACAGATGCTGAGTGTGAAAAATGG
Y221F	GCATCTGTtttCTGCTGATCATTCGGGTTCTG	CAGCAGaaaACAGATGCTGAGTGTGAAAAATGG
L223A	TATCTGgccATCATTCGGGTTCTGTTAAAAGTGG	CGAATGATggcCAGATAACAGATGCTGAGTGTGAAAA
S241A	TTgctCACAGGAAGGCACTGACCACCATCATC	TGCCTTCCTGTGagcAACCCGCAGCCCCGATTC
F260A	TTGTGTgcaCTGCCCTATCACACACTGAGGAC	TAGGGCAGtgcACACAAGAAGAAGATGATCAAGGTGA
