# Supplementary material for: Molecular insights into ago-allosteric modulation at cysteinyl leukotriene receptor 2
Source: Nat Commun. 2025 Dec 16;17:908. doi: 10.1038/s41467-025-67630-7 (PMC12830604; doi:10.1038/s41467-025-67630-7)
Supplement: Supplementary file 2 — Reporting Summary [file 41467_2025_67630_MOESM2_ESM.pdf]

nature portfolio

Corresponding author(s): Heng Liu, Chongyuan Wang

Last updated by author(s): Nov 23, 2025

Reporting Summary

Nature Portfolio wishes to improve the reproducibility of the work that we publish. This form provides structure for consistency and transparency in reporting. For further information on Nature Portfolio policies, see our Editorial Policies and the Editorial Policy Checklist.

Statistics

For all statistical analyses, confirm that the following items are present in the figure legend, table legend, main text, or Methods section.

n/a	Confirmed	
☐	☒	The exact sample size (*n*) for each experimental group/condition, given as a discrete number and unit of measurement
☐	☒	A statement on whether measurements were taken from distinct samples or whether the same sample was measured repeatedly
☐	☒	The statistical test(s) used AND whether they are one- or two-sided<br>*Only common tests should be described solely by name; describe more complex techniques in the Methods section.*
☒	☐	A description of all covariates tested
☒	☐	A description of any assumptions or corrections, such as tests of normality and adjustment for multiple comparisons
☐	☒	A full description of the statistical parameters including central tendency (e.g. means) or other basic estimates (e.g. regression coefficient) AND variation (e.g. standard deviation) or associated estimates of uncertainty (e.g. confidence intervals)
☐	☒	For null hypothesis testing, the test statistic (e.g. F, t, r) with confidence intervals, effect sizes, degrees of freedom and P value noted<br>*Give P values as exact values whenever suitable.*
☒	☐	For Bayesian analysis, information on the choice of priors and Markov chain Monte Carlo settings
☒	☐	For hierarchical and complex designs, identification of the appropriate level for tests and full reporting of outcomes
☒	☐	Estimates of effect sizes (e.g. Cohen's *d*, Pearson's *r*), indicating how they were calculated

Our web collection on statistics for biologists contains articles on many of the points above.

Software and code

Policy information about availability of computer code

Data collection	SerialEM3.7, GROMACS-2023.2
Data analysis	RELION 4.1, cryoSPARC v.4, COOT 0.9.8, PHENIX1.10.1, Chimera1.17.1, ChimeraX1.6.1, Pymol2.5, FlowJo version 10, GraphPad Prism 9.5.0

For manuscripts utilizing custom algorithms or software that are central to the research but not yet described in published literature, software must be made available to editors and reviewers. We strongly encourage code deposition in a community repository (e.g. GitHub). See the Nature Portfolio guidelines for submitting code & software for further information.

Data

Policy information about availability of data

All manuscripts must include a data availability statement. This statement should provide the following information, where applicable:

- Accession codes, unique identifiers, or web links for publicly available datasets
- A description of any restrictions on data availability
- For clinical datasets or third party data, please ensure that the statement adheres to our policy

The cryo-EM density map for the CysLT2R-LTC4-miniGq and CysLT2R-LTD4-miniGq complexes have been deposited in the EMDB under the accession codes EMD-63985 and EMD-63986. The coordinates for the models of CysLT2R-LTC4-miniGq and CysLT2R-LTD4-miniGq have been deposited in the PDB under the accession number 9UAM and 9UAN. All dada needed to evaluate the conclusions in the paper are present in the paper and/or the Supplementary Materials.

Research involving human participants, their data, or biological material

Policy information about studies with human participants or human data. See also policy information about sex, gender (identity/presentation), and sexual orientation and race, ethnicity and racism.

Reporting on sex and gender	N/A
Reporting on race, ethnicity, or other socially relevant groupings	N/A
Population characteristics	N/A
Recruitment	N/A
Ethics oversight	N/A

Note that full information on the approval of the study protocol must also be provided in the manuscript.

Field-specific reporting

Please select the one below that is the best fit for your research. If you are not sure, read the appropriate sections before making your selection.

☒ Life sciences ☐ Behavioural & social sciences ☐ Ecological, evolutionary & environmental sciences

For a reference copy of the document with all sections, see nature.com/documents/nr-reporting-summary-flat.pdf

Life sciences study design

All studies must disclose on these points even when the disclosure is negative.

Sample size	The data size for cryo-EM experiments was determined by the images collected and the particle density in the samples. Sufficient cryo-EM data were collected to achieve the high resolutions of 3D reconstructions. For the functional experiments, the sample size was at least three, adhering to common practice in the field and striking a reasonable balance between statistical robustness and practicality.
Data exclusions	During cryo-EM data processing, it is a common practice to use 3D classifications to remove poor-quality particle images to achieve higher resolution 3D reconstructions.
Replication	Functional experiments and biochemical experiments were repeated at least three times with independent samples, consistently yielding comparable results and reaffirming the reproducibility.
Randomization	This is not relevant to our study and no grouping was needed.
Blinding	Blinding was not implemented in this study, as no subjective allocation was involved in any of the structural and functional experiments.

Reporting for specific materials, systems and methods

We require information from authors about some types of materials, experimental systems and methods used in many studies. Here, indicate whether each material, system or method listed is relevant to your study. If you are not sure if a list item applies to your research, read the appropriate section before selecting a response.

Materials & experimental systems

n/a	Involved in the study	
☐	☒	Antibodies
☐	☒	Eukaryotic cell lines
☒	☐	Palaeontology and archaeology
☒	☐	Animals and other organisms
☒	☐	Clinical data
☒	☐	Dual use research of concern
☒	☐	Plants

Methods

n/a	Involved in the study	
☒	☐	ChIP-seq
☐	☒	Flow cytometry
☒	☐	MRI-based neuroimaging

Antibodies

Antibodies used	mouse anti-Flag-FITC antibody (Proteintech, 66008-4-Ig), scFv16 was derived from the mouse monoclonal antibody mAb16 (DOI: 10.1038/41476-018-06002-w).
Validation	mouse anti-Flag-FITC antibody:https://www.ptgcn.com/products/Flag-tag-Antibody-66008-4-Ig.htm. mouse anti-Flag-FITC antibody is commercially purchased and has been validated by the vendor. Validation data is available from the vendor's website. scFv16 can bind and stabilize the Gi and modified Gg characterized by SDS-PAGE, SEC and Western blots analysis.

Eukaryotic cell lines

Policy information about cell lines and Sex and Gender in Research

Cell line source(s)	HEK-293T(ATCC), Sf9(Expression systems)
Authentication	No further authentication was performed for commercially available cell lines.
Mycoplasma contamination	Periodically test negative.
Commonly misidentified lines (See ICLAC register)	None of the cell lines used are listed in the database of commonly misidentified cell lines maintained by ICLAC.

Plants

Seed stocks	N/A
Novel plant genotypes	N/A
Authentication	N/A

Flow Cytometry

Plots

Confirm that:

- [ ] The axis labels state the marker and fluorochrome used (e.g. CD4-FITC).
- [ ] The axis scales are clearly visible. Include numbers along axes only for bottom left plot of group (a 'group' is an analysis of identical markers).
- [ ] All plots are contour plots with outliers or pseudocolor plots.
- [ ] A numerical value for number of cells or percentage (with statistics) is provided.

Methodology

Sample preparation	HEK293T cells expressing Flag-tagged GPR99 were harvested 24 h post-transfection. Cells were resuspended in PBS buffer, blocked with 5% (w/v) BSA at room temperature for 30 min, and incubated with mouse anti-Flag-FITC antibody (Proteintech) at a 1:200 dilution for 30 min at 4°C. After washing with PBS buffer, surface expression was quantified by detecting FITC fluorescence intensity using a LSR Fortessa flow cytometer (BD Biosciences). FACS data were analyzed with FlowJo version 10. Approximately 20,000 cellular events per sample were collected.
Instrument	a LSR Fortessa flow cytometer (BD Biosciences)
Software	FlowJo version 10
Cell population abundance	Approximately 20,000 cellular events were collected and the mean fluorescence intensity of positive expression cell population was calculated.
Gating strategy	Gating was determined by the fluorescent intensity of FITC to differentiate positive cells and all other cell

- [ ] Tick this box to confirm that a figure exemplifying the gating strategy is provided in the Supplementary Information.
